# Restricting Lower Limb Flail is Key to Preventing Fatal Pelvic Blast Injury

**DOI:** 10.1007/s10439-019-02296-z

**Published:** 2019-05-30

**Authors:** Iain A. Rankin, Thuy-Tien Nguyen, Diagarajen Carpanen, Jonathan C. Clasper, Spyros D. Masouros

**Affiliations:** The Royal British Legion Centre for Blast Injury Studies, Department of Bioengineering, Imperial College London, South Kensington Campus, London, SW7 2AZ UK

**Keywords:** Biomechanics, Pelvis, Fracture, Vascular injury, Traumatic amputation

## Abstract

Pelvic vascular injury in the casualty of an explosive insult is a principal risk factor for increased mortality. The mechanism of injury has not previously been investigated in a physical model. In this study, a small-animal model of pelvic blast injury with a shock-tube mediated blast wave was utilised and showed that lower limb flail is necessary for an unstable pelvic fracture with vascular injury to occur. One hundred and seventy-three cadaveric mice underwent shock-tube blast testing and subsequent injury analysis. Increasingly displaced pelvic fractures and an increase in the incidence of pelvic vascular injury were seen with increasing lower limb flail; the 50% risk of vascular injury was 66° of lower limb flail out from the midline (95% confidence intervals 59°–75°). Pre-blast surgical amputation at the hip or knee showed the thigh was essential to result in pelvic displacement whilst the leg was not. These findings, corroborated by clinical data, bring a paradigm shift in our understanding of the mechanism of blast injury. Restriction of lower limb flail in the human, through personal protective equipment, has the potential to mitigate the effects of pelvic blast injury.

## Introduction

The rise of the improvised explosive device as the weapon of choice in recent conflicts has changed the nature of injuries from gunshot wounds to the extensive tissue loss associated with blast injury.^21^ One of the most severe patterns of injury associated with these weapons is the dismounted complex blast injury, which is an explosion-induced battlefield injury sustained by a soldier on foot patrol (dismounted). It involves traumatic amputation of at least one lower extremity, a severe injury to another extremity, and pelvic, abdominal or urogenital wounding.^5^

A leading risk factor for increased mortality in dismounted blast injury is that of pelvic fracture, with an associated mortality of 60.8% compared to 22.9% in casualties without a pelvic fracture.^30^ Pelvic fracture patterns are of a mechanically unstable nature, characterised by an open pelvic fracture with disruption at the pubic symphysis (PS) and sacroiliac (SI) joints.^29^ The most common cause of death in dismounted pelvic blast injury is exsanguination, with uncontrolled haemorrhage from proximal large vessel trauma.^13^,^24^ Data from the UK Joint Trauma Theatre Registry (JTTR) identified 59 military blast fatalities from recent conflicts that sustained pelvic fracture without more severe injuries to other body regions (higher ISS score remote to the pelvis). The cause of death was identified as a pelvic vascular injury in all cases.^28^

The mechanism by which blast in the dismounted casualty leads to pelvic vascular injury is not known. Post-mortem CTs of UK JTTR blast casualties with pelvic fractures have been reviewed to correlate pelvic bony displacement with vascular injury. Receiver operating characteristic (ROC) curve analyses performed assessed the sensitivity and specificity of the different directions of pelvic displacement at the PS and SI joints, measuring distance of disruption as a predictor of vascular injury. A significant association with vascular injury was seen with any degree of lateral displacement of the SI joints, the displacement distance of which was found to have the highest sensitivity and specificity of any directions for predicting vascular injury, with an area under the curve value of 0.73.^28^

Lower limb flail has been hypothesised as the mechanism of injury in blast-mediated traumatic amputation. The original theory described the blast wave coupling with long bones, causing diaphyseal fracture through axial and shear stresses, followed by the limb flailing from the blast wind to complete the traumatic amputation at the level of the fracture.^7^ More recent data has suggested lower-extremity flail in isolation as a valid traumatic amputation mechanism, to account for the higher rate of through joint traumatic amputations seen compared to historic data (24.1 vs 1.3%) and the lack of a link between primary blast lung injury and traumatic amputation, as had previously been observed.^23^ Pelvic fracture strongly correlates with traumatic amputation; it occurs more frequently in those with bilateral lower limb amputation than those with unilateral lower limb amputation, and is also significantly associated with a higher level of traumatic amputation.^30^ Of the fifty nine pelvic fatalities identified from the UK JTTR, 92% had also sustained a traumatic amputation.^28^ The significant correlation between traumatic amputation and dismounted pelvic blast injury suggests they may share the same mechanism of injury. This mechanism of injury has not previously been investigated in a dismounted pelvic blast physical model.

The purposes of this study were (1) to replicate dismounted pelvic blast injury in a small animal model utilising a shock tube mediated blast wave and (2) to investigate the effects of restricting lower extremity flail on mitigating the severity of pelvic bony and vessel injury. Our hypothesis was that unstable, displaced pelvic fracture patterns and pelvic vascular injury are associated with blast-wave mediated lower limb flail.

## Materials and Methods

**Figure 1:**
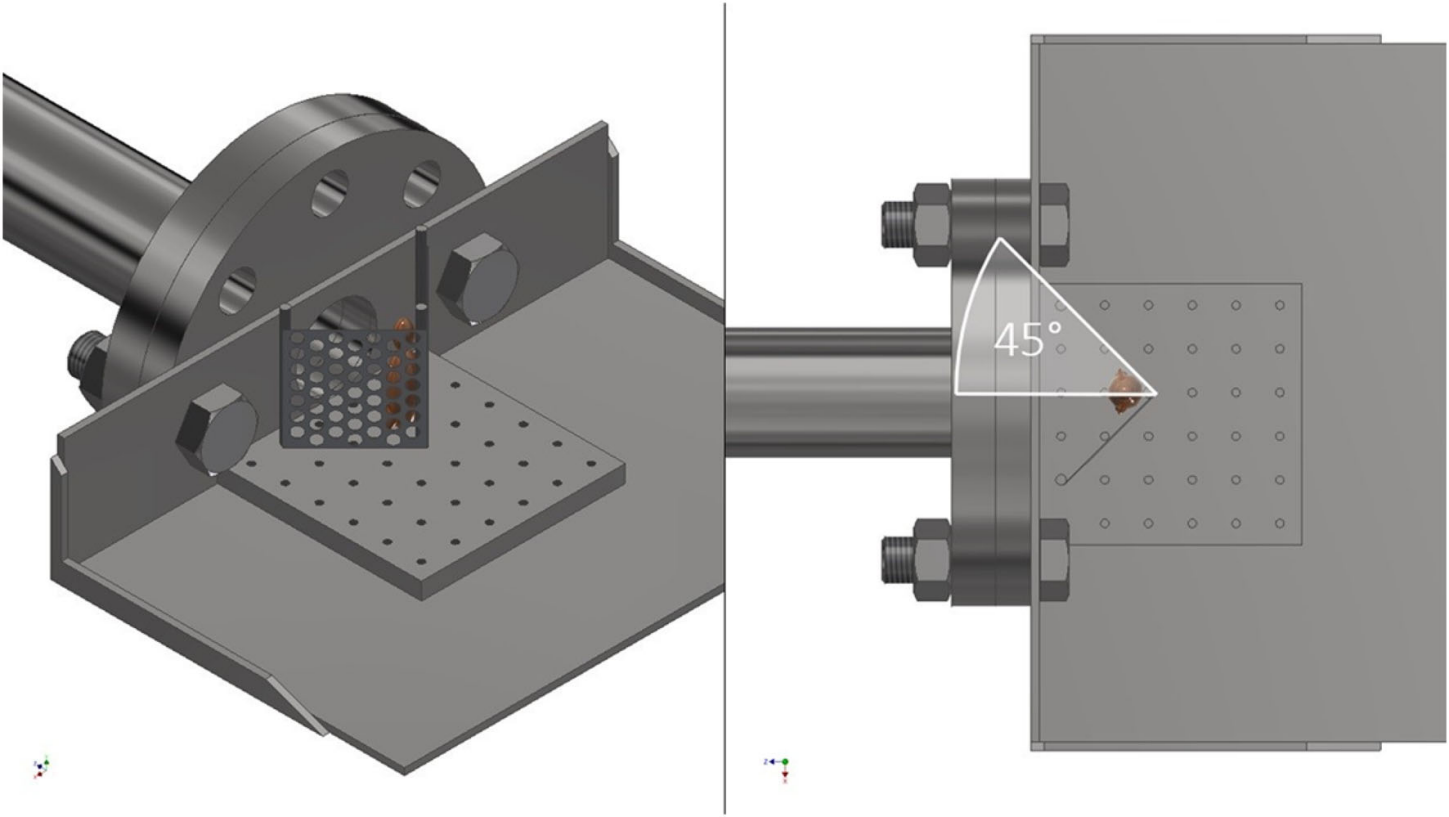
(a) Left: Shock tube with mounting platform, fenestrated steel fence restricting lower limb flail, and position of mouse (represented with model). (b) Right: Aerial view with restriction of lower limb flail to 45° group, demonstrating the angle as measured from the midsagittal plane.

Animals experimental design and procedures were carried out in compliance with the UK Animal (Scientific Procedures) Act 1986. Shock tube testing was conducted on fresh-frozen cadaveric male MF-1 (outbred, ex-breeder, wild type) mouse specimens (8–9 weeks of age, Charles River Ltd, UK). Specimens were stored at − 20°C and subsequently thawed at room temperature (21 ± 2°C) prior to testing. Mice were secured on a stainless-steel platform, distal to the outlet flange of a double diaphragm shock tube.^12^ Three cable ties were applied to secure specimens in position—across the abdomen, thorax and neck—whilst allowing free range of motion of the lower extremities. A fenestrated steel fence was attached to the platform’s central restraint, positioned at varying angles from the midline, to restrict outward flail of the lower extremity (Fig. 1a). The main cohort of 103 mice had tests conducted with the steel fence at 45°, 60°, 90°, 105°, 135°, and unrestricted (180°), as measured of each side from the midsagittal plane (Fig. 1b).

Forty mice were assigned to one of three further groups: bilateral amputation at the hip, bilateral amputation at the knee, and bilateral traumatic above knee amputation.

### Bilateral Amputation at the Hip

Mice underwent bilateral lower extremity amputation at the hip prior to testing, to examine the injury pattern when the effects of lower extremity flail are eliminated. Amputation was performed with a posterior approach to the hip joint, disarticulation of the hip, removal of the lower limb and subsequent closure with 4.0 nylon sutures (Ethilon Nylon Suture, Ethicon, New Jersey, USA).

### Bilateral Amputation at the Knee

Amputation at the knee was performed to examine the injury pattern when the effects of the leg upon lower extremity flail are eliminated, as may occur if a below knee traumatic amputation occurred prior to flail of the lower limb. Amputation was performed with a through knee incision made at the patellar tendon, removal of the leg and foot, and subsequent closure with 4.0 nylon sutures. These mice were subsequently tested upon with unrestricted lower limb flail.

### Bilateral Traumatic Above Knee Amputation

The third group of mice sustained a prior crushing injury to both mid-femurs to ensure bilateral traumatic above knee amputations to occur during the blast wave experiments. A small drop tower was utilised to deliver a standardised impact (250 g striker, released from 50 cm height) to a triangular-prism-shaped anvil, placed at the mid-point of the femur.^11^ The anvil was used to produce a crush injury to the soft tissues of the thigh and a transverse femoral shaft fracture, with no disruption in skin continuity. Radiographs were taken post procedure to ensure correct positioning (mid-shaft) of the injury. These mice were subsequently tested upon with unrestricted lower limb flail.

A final thirty mice were tested upon a mobile platform, to ascertain the effects of modifying the boundary conditions of the platform to injuries sustained. The above described mounting platform was positioned within a sliding track, to allow a potential backwards movement from the blast wave of 5 cm.

Pulse evolution along the shock tube was monitored with piezoelectric pressure sensors (Dytran Instruments 2300V1, California, USA) positioned both within and at the outlet flange of the shock tube. Further testing was performed with a sensor at the point of impact of the mouse specimen, to allow shock wave characterisation.

A high-speed digital video camera (Vision Research Phantom v210, Ametek; New Jersey, USA) utilising a vertical view point was used to record and confirm the degrees of movement of the lower extremities in response to the shock wave generated. Images were recorded at 72,000 frames-per-second at a resolution of 128 × 152 pixels.

Following the tests, specimens underwent radiographic imaging (Fluoroscan InSightTM-FD, Hologic Inc., USA) and subsequent dissection to identify pelvic bony and vascular injury. Fractures were classified in accordance with the Tile criteria.^25^ Vessel injuries were confirmed macroscopically during dissection, with 10% of vessel injury samples taken for histological confirmation. Vessel injury was defined as complete transection, with the most proximal large vessel injury noted. Associated traumatic amputation and its level was also noted.

### Statistical Analysis and Development of the Risk Function

NCSS statistical software was used for statistical analysis (Utah, USA). A one-way ANOVA with a Tukey HSD *post hoc* comparison to determine if there were differences in injury type (Tile Type B vs. Type C) between all groups of mice was performed. A Bonferroni corrected *p* value of 0.0083 was used to compensate for multiple comparisons (0.0083 = 0.05/6). Weibull survival analysis was used to examine the association between range (angle from the midline) of lower extremity flail and vascular injury. The amputation and mobile platform subgroups were excluded from this survival analysis. The Weibull regression model is $P(x) = 1 - e^{{ -({x/\lambda})^{\kappa } }},$ where *P* is the probability of injury, *x* is the predictor variable, and *λ* and *κ* are the corresponding coefficients associated with the predictor variable. To derive the survivability curves, data were classified as left censored where vascular injury was present. The normalized confidence interval size (NCIS) of the survivability curves was determined upon the ratio of the width of the CI to the magnitude of the predictor variable, at a specific risk level.

## Results

**Figure 2:**
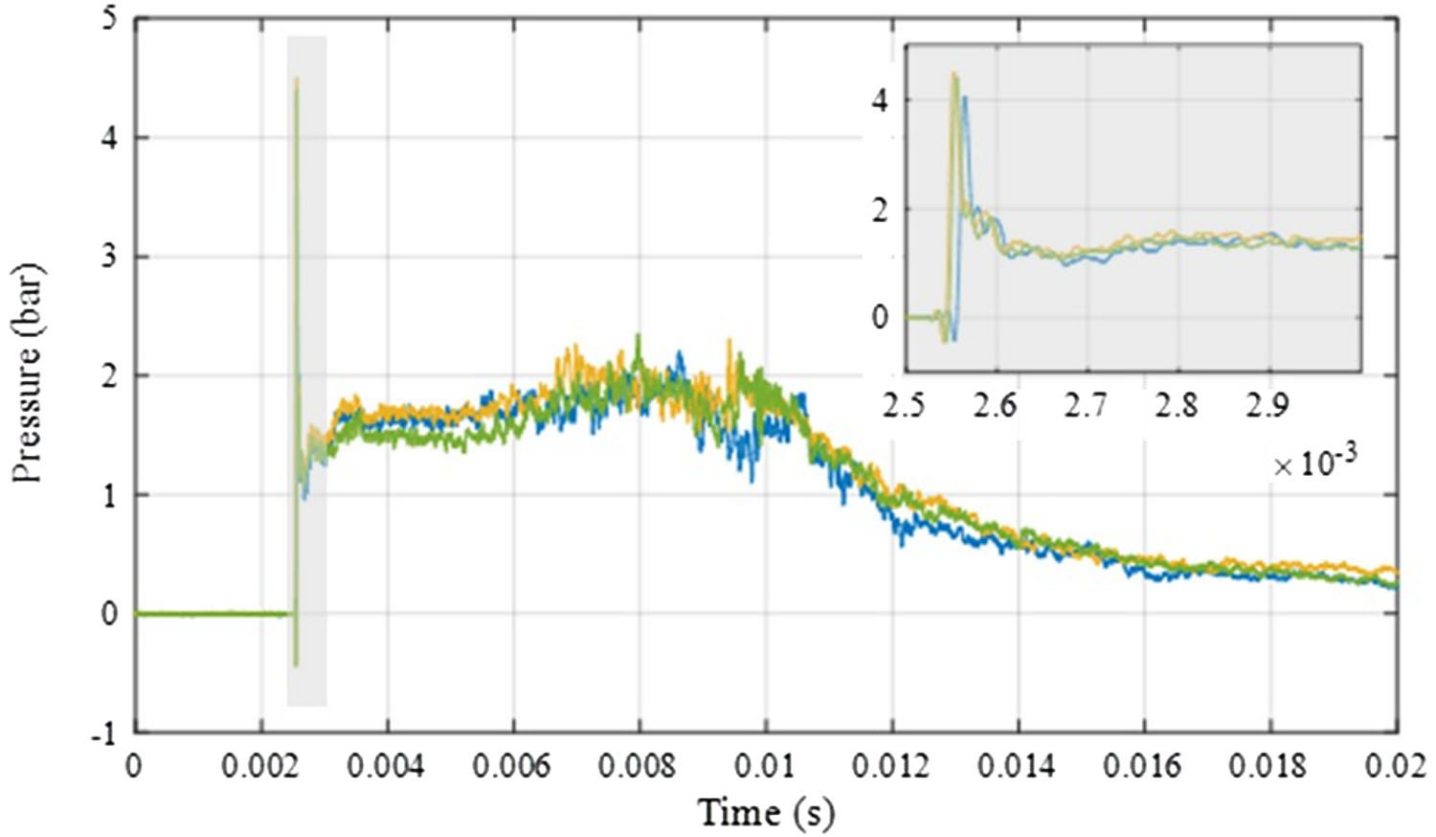
Three repeat blast wave characterisation tests, delivering a maximum peak pressure of 4.28 bar, mean plateau pressure of 1.72 bar, and shock impulse of 24 bar milliseconds.

Preliminary testing and subsequent shock wave characterisation identified a suitable shock wave to allow for pelvic injury, whilst minimising injuries deemed to be non-survivable thoraco-abdominal trauma (Fig. 2).

The incidence of pelvic fractures across all groups was 100%. Pubic symphysis and sacroiliac joint disruption predominated, with an incidence of 82 and 100% respectively. Table 1 details the type of pelvic fractures according to Tile criteria across all mice groups tested and the number of mice allocated to each group.

**Table 1:** Type of pelvic fractures according to Tile criteria across all mice groups.

Mouse group	Number of mice	B1	B2.1	B2.2	B3	C1.1	C1.2	C1.3	C2	C3
Main cohort	103	27	0	0	7	0	43	4	14	8
45° Flail	20	16	0	0	4	0	0	0	0	0
60° Flail	20	3	0	0	1	0	13	0	2	1
90° Flail	20	7	0	0	2	0	7	0	3	1
105° Flail	20	1	0	0	0	0	15	1	2	1
135° Flail	20	0	0	0	0	0	6	3	7	4
180° Flail	3	0	0	0	0	0	2	0	0	1
Subgroups										
Amputated at hip	20	14	0	0	6	0	0	0	0	0
Amputated at knee	10	0	0	0	0	0	2	0	7	1
Above knee TA group	10	0	0	0	0	0	3	0	6	1
Moving platform 45°	10	9	0	0	1	0	0	0	0	0
Moving platform 90°	10	0	0	0	0	0	7	0	3	0
Moving platform 135°	10	0	0	0	0	0	1	0	8	1

TA, traumatic amputation

A one-way ANOVA showed statistically significant differences between the main cohort groups with regards to fracture types (F-value = 29.16, DF = 5, *p* < 0.0001). A Tukey HSD *post hoc* comparison test revealed significant differences between the 45° group, who sustained entirely Type B injuries, and all other groups. Significant differences also existed between the 90° group and the 105° and 135° groups, due to a higher proportion of Type B injuries within the 90° group.

Vessel injury was present in 73 (71%) of the 103 mice within the main cohort. Table 2 details the incidence of vessel injury across the different degrees of restriction of lower extremity flail in the main cohort.

**Table 2:** Incidence and location of vessel injury across main cohort.

Mouse group	Number of mice	Vessel injury	Aorta	Common iliac	External iliac	Internal iliac
45° flail	20	0 (0)	0 (0)	0 (0)	0 (0)	0 (0)
60° flail	20	14 (70)	0 (0)	7 (50)	5 (37)	2 (13)
90° flail	20	14 (70)	0 (0)	0 (0)	10 (71)^a^	5 (36)^a^
105° flail	20	20 (100)	6 (30)	9 (45)	5 (25)	0 (0)
135° flail	20	20 (100)	12 (60)	8 (40)	0 (0)	0 (0)
180° flail	3	3 (100)	0 (0)	3 (100)^a^	1 (33)	0 (0)
Total	103	73 (71)	18 (24)	28 (38)^a^	22 (30)^a^	8 (11)^a^

Results presented as numerical value (%)

^a^Bilateral injuries

**Figure 3:**
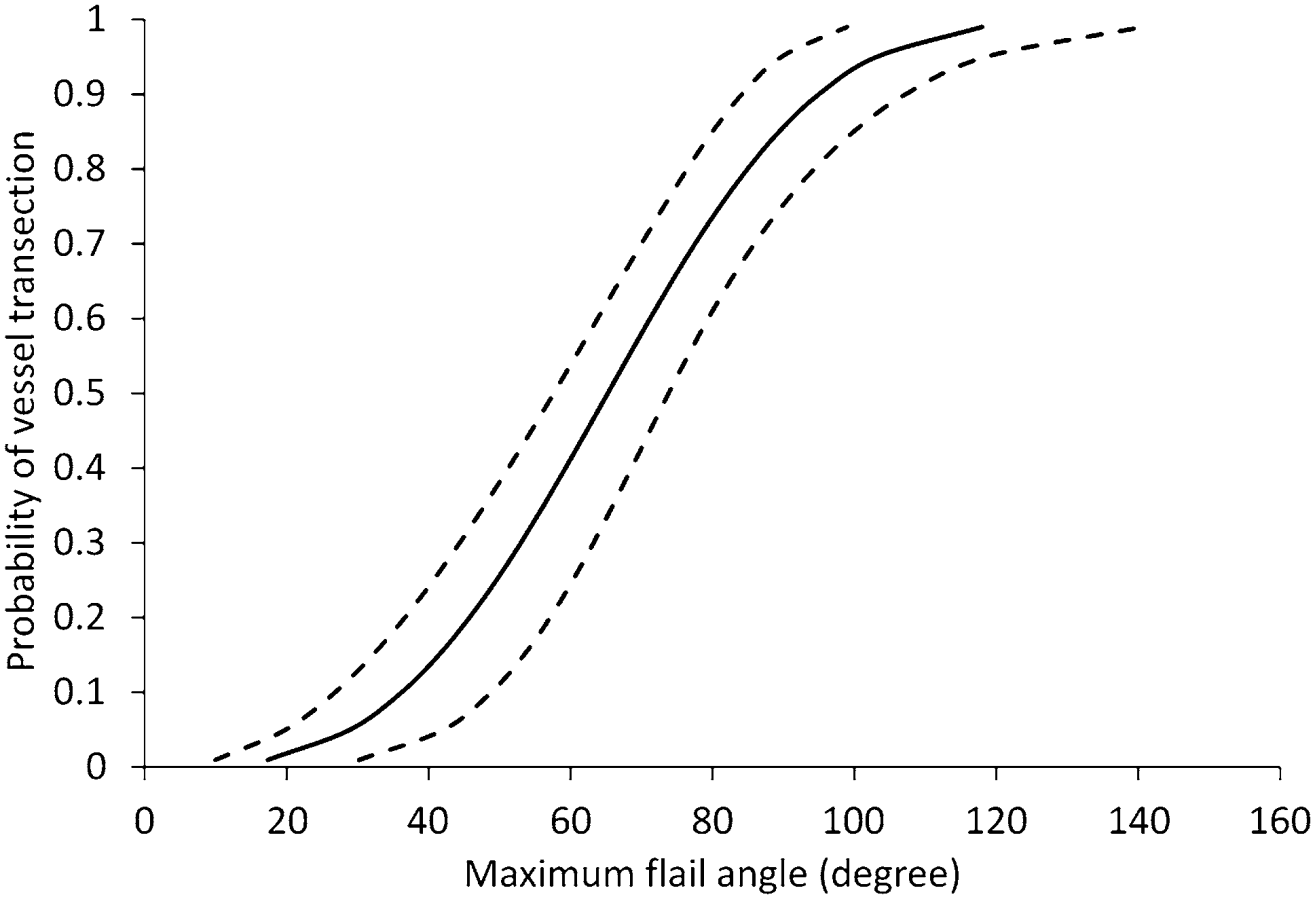
Vascular injury risk curve as a function of maximum allowable angle of lower extremity flail.

Histology confirmed arterial and venous vascular tissue in all samples obtained. Vessel injury increased with increasing lower extremity flail; the 50% risk of vessel injury associated with lower extremity flail was 66° (95% CI 59°–75°) with a low NCIS of 0.24. The full injury risk curve with associated 95% CI is shown in Fig. 3.

No mice with amputation at the hip sustained a vessel injury. There was a 100% incidence of vessel injury within the amputation at the knee group and a 100% incidence of vessel injury in the traumatic above knee amputation group.

No significant differences were seen in the incidence of vessel injury when comparing the mobile platform groups (45°, 90°, 135°) with the non-mobile equivalent groups.

Nine mice (8%) across the main cohort sustained a traumatic amputation. This was at the level of the hip in three (3%), femur in two (2%), and tibia in four (4%). All mice which received a pre-test crush injury to the femoral midshaft went on to sustain bilateral traumatic amputations at the level of the femur.

## Discussion

This study replicated dismounted pelvic blast injury in the mouse model, via a shock-tube mediated blast wave. It is the first study to investigate a physical model of dismounted pelvic blast injury. We hypothesise that lower extremity flail transfers loads which result in lateral displacement and external rotation of the hemipelvis. This, in combination with the blast wave impacting directly upon the pelvis, generates unstable, displaced pelvic ring fractures. Pelvic bony displacement, and subsequent displacement of the intra-pelvic soft tissues, is thought to be responsible for tension on the vasculature and subsequent rupture. An association of vascular injury to increasing lower extremity flail was seen in this study. The injury curve presented (Fig. 3) displays a 50% probability of vascular injury at 66° of lower extremity flail, with a low NCIS. It shows a clear link between increasing angle of lower extremity flail and major vessel injury.

Battlefield data has shown unstable pelvic fracture patterns consisting of PS and SI joint disruption, with posterior pelvic bleeding, as characteristic of the most severe pattern of dismounted blast injury.^13^,^28^,^29^ Our model has reproduced this pattern of injury with PS and SI disruption, unstable fracture patterns and vascular injury occurring posteriorly at the bifurcation of the external and internal iliacs.

**Figure 4:**
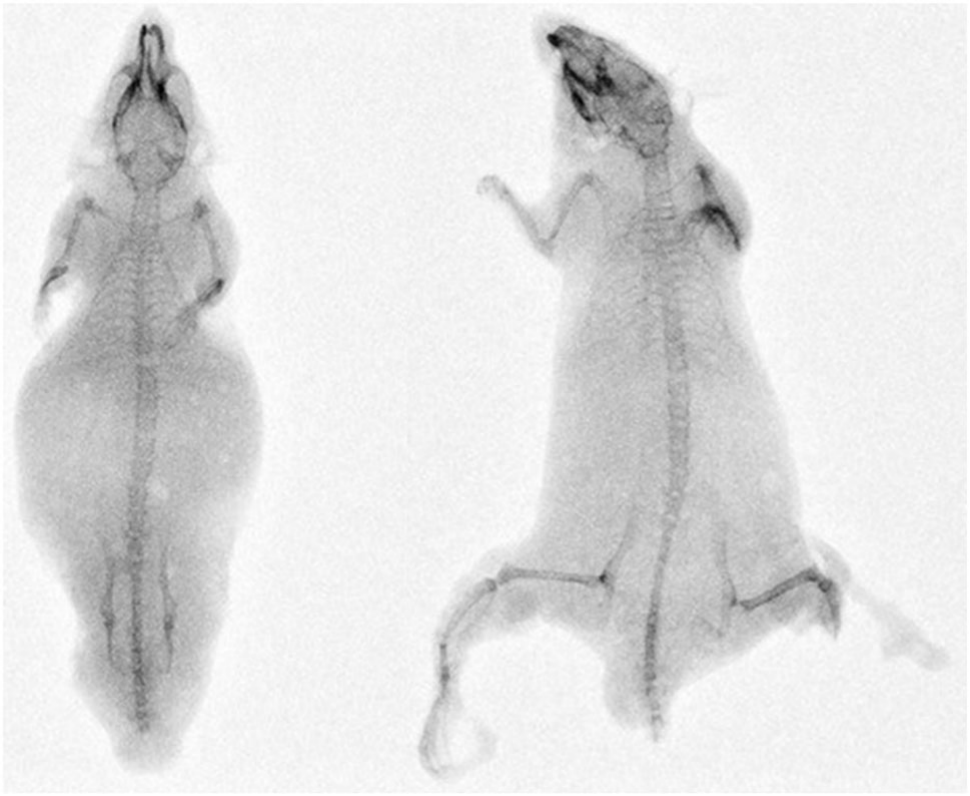
(a) Left: Lower extremity amputated mouse with pubic symphysis disruption, pubic rami fractures and left sided sacroiliac joint disruption. (b) Right: 135° lower extremity flail mouse with pubic symphysis disruption, pubic rami fractures and bilateral sacroiliac joint disruption.

The mice with iatrogenic lower extremity amputation at the hip presented a cohort for which the effects of lower extremity flail were removed. These mice developed exclusively Tile type B fractures, which were minimally displaced (Fig. 4a). They were subsequently found to have no vascular injury. This was similar to the mice restricted to 45° of lower extremity flail and in contrast to the mice seen at higher degrees of lower extremity flail. The latter groups sustained vascular injury with Tile type C fractures, which were significantly displaced (Fig. 4b).

These findings, corroborated by battlefield data,^13^,^28^,^29^ suggest that limb flail is likely a cause for an unstable pelvic fracture with posterior pelvic bleeding to occur in dismounted victims of an explosive insult.

Vessel injury was defined as complete transection of a major vessel. In several specimens, no vessel injury was found. All mice identified as having no vessel injury were explored to confirm uninjured arterial and venous vasculature. Small vessel injury may have been missed due to the use of cadaveric mice, where active bleeding was not observed. As such the conclusions drawn from this paper focus on major vessel injury, such as that of the common, external or internal iliac vessels. In cases of severe disruption of the arterial tree, proximal to the pelvic vasculature, the aorta was noted as the most proximal vascular injury. In the remainder of cases, vessel transection and complete discontinuity was noted to have occurred in the immediate vicinity of the bifurcation of the common iliac artery. 10% of samples were taken to confirm vascular type. In all histological samples the presence of both arterial and venous tissue was noted, suggesting that disruption of both the arterial and venous vasculature had occurred.

In this study a shock tube replicated the effects of a primary blast wave but has not replicated the effects of secondary (such as soil projectile) or tertiary (such as body impact with structures) blast injury. It is hypothesised these effects would compound the injuries seen. The lower traumatic amputation rates seen in this study are similarly also thought to be due to the absence of these effects.

Traumatic amputation has previously been hypothesised to occur due to the blast wave coupling with long bones with a resultant diaphyseal fracture, before limb flail completes the amputation.^7^ This was not observed in this study, at rates which match the degree of traumatic amputation seen in association with pelvic fracture as noted from battlefield data.^30^ In agreement with other authors, limb flail is felt necessary for traumatic amputation to occur.^7^,^23^ The lower rates of traumatic amputation seen in this study may be hypothesised to be due to a blast wave of inadequate pressure to generate diaphyseal fracture, or due to the absence of secondary blast injury causing an initial disruption to the soft tissues of the thigh. At higher loading conditions the threat was felt to be non-survivable, due to thoraco-abdominal injury, and so this was thought unlikely to be the cause. All mice which had sustained a pre-test crush impact to the thigh went on to sustain above knee traumatic amputations, suggesting an element of injury in addition to the flail mechanism is required. These mice also sustained displaced pelvic fractures with vascular injury. This contrasts with the mice amputated at the hip, who sustained no pelvic displacement or vascular injury. These findings suggest that the process of pelvic displacement and vascular injury occur during flail, prior to above knee traumatic amputation, whilst the femur is still able to transfer loads resulting in lateral displacement and external rotation of the pelvic girdle. Mice which received pre-test through knee amputations similarly went on to display displaced pelvic fractures with vascular injury, suggesting that the thigh—but not the leg—is the essential component in lower limb flail causing injury. This may account for the high rate of above knee traumatic amputations, but not below knee amputations, seen in association with pelvic blast injury.^10^,^30^ Our findings suggest that above knee amputation may happen in conjunction with dismounted pelvic blast injury, but that below knee amputation may occur through an unrelated injury mechanism.

Preliminary testing indicated injury to occur at lower flail angles than unrestricted (180°) flail. As such the unrestricted flail group within the main cohort was not continued beyond the preliminary three mice. Experiments with a mobile platform were performed to ascertain if changing the boundary conditions would alter injuries seen. No differences were seen in the rates of vascular injury across the mobile and stationary platform groups.

The mouse was chosen as a suitable animal model due to its similarities to the human pelvis at the areas of key interest from battlefield data: the pubic symphysis, sacroiliac joints and pelvic vasculature. They are one of the few animal models with a fibrocartilaginous pubic symphysis,^1^,^14^ whilst the sacroiliac joints are a recognised animal model in the investigation of sacroiliac joint disease.^6^,^16^,^22^,^26^,^27^ The pelvic vasculature of the mouse follows that of the human; the common iliac artery arises from the aorta before branching to give the internal and external iliac arteries. This contrasts with other animals whereby the external and internal iliac arteries arise directly from the aorta.^8^

Several factors must be taken into consideration when inferring the results and conclusions of these findings in the mouse model, for subsequent interpretation to human injury risk. The main morphological differences of the mouse to the human pelvis are that mouse ilia are larger in the axial plane, whilst shorter in the sagittal and coronal planes. Furthermore, mouse pubic rami are shorter compared to those of humans.^26^ The human pelvis is therefore comparatively compressed in the axial plane whilst elongated in the sagittal and coronal planes, having evolved to aid bipedal locomotion.^9^ Despite these morphological differences, the conclusions of this study focus solely on outward flail of the lower limb as the mechanism of injury. In the upright posture as in our study, the angle of rotation is similar between the species. As explored in the amputated mice, it is the outward motion of the femur causing a lateral displacing force upon the pelvis, which results in displaced fractures and vascular injury. As such, the morphological differences of the pelvis between species is minimised when focusing upon this mechanism of injury and conclusion. Further consideration must also be given to the respective forces of the mouse femur, and that of the human femur, acting upon the pelvis. The mouse femur accounts for 15.1% of total skeletal length, whilst the human femur is comparatively heavier and accounts for 26.7% of total skeletal length.^3^,^4^ As such, a proportionally greater moment is expected to act upon the human pelvis during blast compared to the mouse. It is unclear how this would alter the injury curve, however, as the human pelvis may have proportionally greater strength to resist this greater moment. It is uncertain therefore how these data scale to the human. Irrespective of scaling, this study has shown that limitation of lower limb flail mitigates the severity of pelvic injury and likelihood of vascular injury in the mouse model, and so a similar effect would be expected in the human.

Scaling laws for small animal models in blast consider factors including force, mass and velocity, but no scaling laws exist for translating angle of flail, as in this study.^15^ Blast tolerance in small and large animal models, and how it scales to the human, has been described previously in relation to traumatic blast lung injury (TBLI).^2^ The lethal median dose (LD_50_), causing fatal TBLI in the mouse is reached at shock-tube plateau pressures of 29.0–30.7 psi with durations of 3–6000 ms.^17^–^20^ The shock wave in this study delivered a mean plateau pressure of 1.72 bar (24.9 psi) sustained over 7.6 ms. The maximum peak pressure in this study was 4.28 bar (62 psi) and sustained for less than 0.008 ms. Due to the short duration, this falls below any possible LD_50_ estimations from injury curves derived from the above studies.^19^ As such, our shock wave was thought to be representative of a blast wave falling below the LD_50_ observed in previous studies to cause fatal TBLI, but a sufficient insult to cause pelvic vessel transection due to lower limb flail.

Reproducing blast mediated lower limb flail in the human is challenging due to the limitations of free field blast tests and the suitability of shock tubes only for small animal models.^11^,^12^ Future research strategies are required to overcome the obstacles of free field blast tests, or progress dismounted blast-injury research through the development of larger shock tubes to simulate the dismounted blast environment with human cadavers. Computational modelling could be used in combination with the results from this study to assess the angle of flail and the loading required to result in pelvic vascular injury in the human pelvis. We would speculate a starting point in any future research to adopt a scaling of 1:1, until further data corroborates or indicates otherwise. Although it is unclear how these data scales to the human, one factor is clear: limitation of lower limb flail mitigates the effect of pelvic injury in this mouse model. Any restriction of flail in the human, through military personal protective equipment, would be beneficial to mitigate the effects of dismounted pelvic blast injury.

This study is the first to replicate dismounted pelvic blast injury using a small animal model. Our results suggest that lower limb flail is necessary for an unstable, fatal pelvic fracture to occur. Restriction of lower limb flail was shown to reduce the probability of vascular injury, and therefore of mortality. An injury-risk curve was developed which associates restriction of lower limb flail to the probability of vascular injury; restriction to 66° flail results in a 50% probability of vascular injury. Scaling these angles of restriction to the human is unclear, however, any degree of restriction would be beneficial in mitigating the effects of injury. These findings determine the mechanism of injury to the lower body in dismounted blast and suggest a mitigation strategy not previously considered. Limitation of lower limb flail in the next generation of personal protective equipment may reduce the high mortality rates associated with dismounted pelvic blast injury.
